# Relationships between Sprint Ability and Endurance Capacity in Soccer Referees

**DOI:** 10.3390/sports6020028

**Published:** 2018-03-30

**Authors:** Mario Sánchez-García, Javier Sánchez-Sánchez, Alejandro Rodríguez-Fernández, David Solano, Daniel Castillo

**Affiliations:** 1 Research Group Planning and Assessment of Training and Athletic Performance, Pontifical University of Salamanca, 37002 Salamanca, Spain; mario.sanchez@ui1.es (M.S.-G); jsancheza@upsa.es (J.S.-S); david_lolo_2907@hotmail.es (D.S.); 2 Faculty of Health Sciences, University Isabel I, 09003 Burgos, Spain; alejandro.rodriguez.fernandez@ui1.es

**Keywords:** match officials, associations, speed, resistance

## Abstract

The aim of this study was to analyze the association between sprint ability and endurance capacity in soccer referees. Twenty-three Spanish officials participated in this study. Each referee undertook, in this order, a 40 m linear straight sprinting test (40 m Sprint) and the Yo–Yo intermittent recovery level 1 test (YYIR1) interspersed with a 8 min of self-administered rest. The results in the 40 m Sprint test showed that the time spent by referees was 5.56 ± 0.27 s and achieved a maximum velocity of 31.46 ± 2.85 km·h^−1^. Furthermore, during the YYIR1 the referees covered 1213.91 ± 432.26 m. The distance covered at YYIR1 was moderately correlated to the velocity achieved in the 40 m Sprint test (r = −0.404, *p* < 0.05). These results suggest that the ability to reach high speeds is a limiting factor in YYIR1 performance.

## 1. Introduction

Soccer refereeing is a high demanding activity in terms of the higher total distance covered and sprints bouts performed [1]. In this sense, soccer referees cover approximately 10 km, of which 2.7 km are covered at high speeds (>18 km·h^−1^) during the Spanish National Division [2,3,4]. Likewise, they undertook 21.3–30.5 sprints at a speed above 25.2 km·h^−1^ [1]. Besides, around 29 km·h^−1^ is the maximum velocity registered by referees during official matches [4]. Attending to these high physical match demands, the challenge of the physical coaches of match officials is to ensure training programs by keeping up with play at all times to attain optimal positioning when making key decision [1]. Most of scientific studies have analyzed the association between match performances and fitness variables with the aim of establishing the battery test requirement by the National and International Soccer Referees Committees [5,6,7]. In the early stages of the fitness battery for match officials, they had to pass 3 specific tests (2 × 200-m sprint, 2 × 50-m sprint and 12-min run test), even though these tests were, in hindsight, poorly associated with match play activities [5]. New fitness tests, such as the sprinting test and a cardiovascular fitness test, have been recently added to the fitness battery for match officials [8]. However, the relationships among different performances in field tests has been less investigated [9,10]. Thus, it would be interesting to study the relationships between field tests (i.e., sprint ability and endurance capacity) in order to design appropriate physical training programs. 

Therefore, the aim of this study was to analyze the association between sprint ability (i.e., sprint 40 m test) and endurance capacity (i.e., Yo–Yo Intermittent Recovery test level 1) in soccer referees.

## 2. Methods

### 2.1. Participants

Twenty-three Spanish officials (24 ± 5 years, 179 ± 5 cm, 74.7 ± 9.8 kg) participated in this study from the Committee of Soccer Referees in Castilla y León (Spain). Each referee had at least 6 years of refereeing experience. All match officials trained at least three times a week and were involved in refereeing on average three times per month. Referee fulfilled the following inclusion criteria: (1) a background of ≥4 years of systematic training and competitive experience, (2) continuous training for the previous 3 months with absence of musculoskeletal injury, (3) absence of potential medical problems that could compromise participation or performance in the study, and (4) absence of any lower-extremity surgery in the past 2 years. Participants were informed of the experimental design and signed an informed consent form prior to the investigation. This investigation was performed in accordance with the Declaration of Helsinki.

### 2.2. Measures and Procedures

Referees performed a standard warm-up consisting of 7 min of slow jogging followed by progressive sprints and dynamic stretching. Each referee undertook, in this order, a 40 m linear straight sprinting test (40 m Sprint) and the Yo–Yo intermittent recovery level 1 test (YYIR1) interspersed with a 8 min of self-administered rest [11].

The 40 m Sprint consisted of two maximal sprint trials of 40 m length interspersed with a 90 s rest period between sprints. This test was performed on an outdoor athletics track and referees were equipped with global positioning system device (Polar Team Pro v.2.0., Polar^®^, Kempele, Finland) operating at the sampling frequency of 10 Hz. Participants’ starting position was 0.5 m behind the first timing gate (WittySEM, Microgate^®^, Bolzano, Italy) and they were asked to run as fast as possible over 40 m. The best time and the maximum velocity achieved during this test were registered for further analyses.

The YYIR1 consisted of 2 × 20 m runs back and forth between two lines at a progressively increasing speed controlled by audio bleeps from a CD (compact disc). Each bout was interspersed with a 10 s of active rest period consisting of 2 × 5 m of jogging. When the participants twice failed to reach the corresponding line in time, the distance covered was recorded and represented the test result [12,13]. 

### 2.3. Data Analysis

Results are presented as means ± standard deviations (SD). The normal distribution of results of the variables applied was tested using the Kolmogorov-Smirnov test, and statistical parametric techniques were conducted. Relationships between the referees’ fitness capacities were examined using Pearson’s product-moment correlation coefficient (r), with 95% confidence intervals (CI) [4]. To interpret the results the threshold values for Pearson product-moment used by Salaj and Markovic [14] were used: low (r ≤ 0.3), moderate (0.3 < r ≤ 0.7) to high (r > 0.7). Data analysis was performed using the Statistical Package for Social Sciences (version 21.0 for Windows, SPSS^®^ Inc., Chicago, IL, USA). Statistical significance was set at *p* < 0.05.

## 3. Results

The results in the 40 m Sprint test showed that the time spent by referees was 5.56 ± 0.27 s and achieved a maximum velocity of 31.46 ± 2.85 km·h^−1^. Furthermore, during the YYIR1 the referees covered 1213.91 ± 432.26 m.

**Figure 1:**
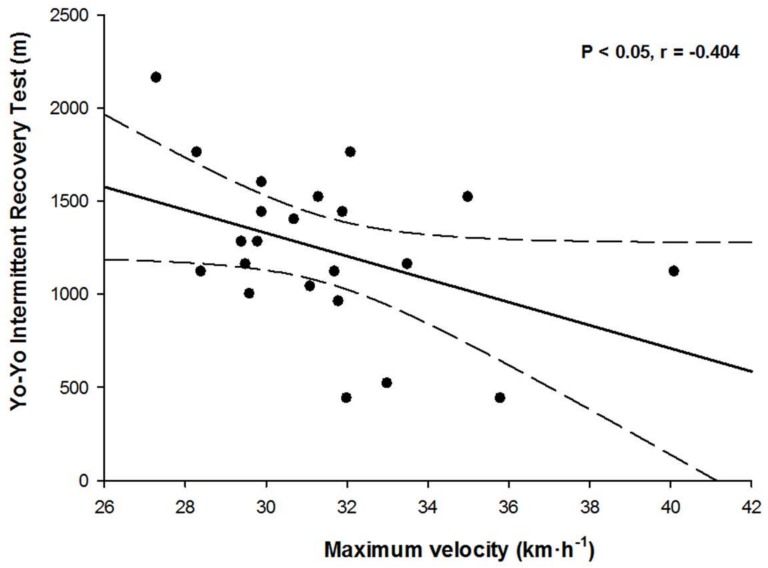
Relationship between maximum velocity achieved in a 40 m Sprint test and distance covered in Yo–Yo Intermittent Recovery test level 1 (YYIR1).

The distance covered at YYIR1 was moderately correlated to the maximum velocity achieved in the 40 m Sprint test (r = −0.404, *p* < 0.05, Figure 1). However, no significant correlations was found between the time to cover 40 m Sprint test and the distance covered at YYIR1 (r = −0.178, *p* > 0.05) and velocity achieved in this 40 m Sprint test (r = −0.226, *p* > 0.05).

## 4. Discussion

The main objective of this study was to analyze the association between sprint ability (i.e., 40 m Sprint test) and endurance capacity (i.e., YYIR1). The results pointed out that there is a moderate correlation between the maximum velocity achieved for the referees and the distance covered in an endurance test. 

Some authors have demonstrated that sprint bouts lasted approximately 4 s during matches [15]. It seems interesting to assess sprinting tests lasting close to 4 s in soccer referees. The results obtained in our study (5.57 ± 0.27 s) were similar to those observed with professional Chilean (5.32 ± 0.01 s) [16], with top class Federation of International Football Association (FIFA) licensed (5.70 ± 0.17 s) [17] and with professional English (5.59 ± 0.21 s) [7] referees. Otherwise, the performance in the YYIR1 test was better in top level referees (1874 ± 431 m and 1743 ± 596 m) [9,11] than in our study (1213 ± 432 m). Thus, it seems that the endurance capacity is determinant to officiate at a higher standard of play. 

Attending to the relationship between the sprint and endurance tests, this study demonstrated that the velocity achieved in the 40 m Sprint test has a moderate relation with YYIR1 (r = −0.404, *p* < 0.05, Figure 1). Therefore, it may be possible that the structure of the YYIR1 and the sprinting bouts has a relation with the speeds requirements in this test. Likewise, this moderate correlation could be due to the intermittent endurance capacity which allow to increase the performance high-intensity repeated actions [18]. However, due to no association was found between the performance in the 40 m Sprint and the velocity achieved in this linear straight test could be because it is mainly influenced by the acceleration capacity. In these sense, the maximum speeds are achieved in the distance of 30–40 m [19]. Even though the distance covered in each serial during the YYIR1 is 40 m, the running patterns follow the sequence 20 + 20 m. Due to the running patterns the participants have to perform continuous accelerations and decelerations, which suppose a neuromuscular fatigue different to that observed in the linear efforts [20].

Taking into account this issue, it would be interesting to assess the acceleration (i.e., 5–10 m sprint distance) ability because the structure of the YYIR1 requires evaluating the short-term actions at high-intensity. Besides, further research is necessary to determine the correlations in match officials.

## 5. Conclusions

Due to the moderate association reported between the maximum velocity achieved in a sprint test and the distance covered in the endurance test, it seems that the ability to reach high speeds is a limiting factor in YYIR1 performance.
